# Identification of nasopharyngeal carcinoma metastasis-related biomarkers by iTRAQ combined with 2D-LC-MS/MS

**DOI:** 10.18632/oncotarget.9067

**Published:** 2016-04-27

**Authors:** Zhen Chen, Lu Long, Kun Wang, Facai Cui, Lepan Zhu, Ya Tao, Qiong Wu, Manlin Xiang, Yunlai Liang, Shiyang Qiu, Zhiqiang Xiao, Bin Yi

**Affiliations:** ^1^ Department of Clinical Laboratory, Xiangya Hospital, Central South University, Changsha, Hunan Province, China; ^2^ The Higher Educational Key Laboratory for Cancer Proteomics and Translational Medicine of Hunan Province, Xiangya Hospital, Central South University, Changsha, Hunan, China

**Keywords:** nasopharyngeal carcinoma, metastasis, iTRAQ, TRIM29, SQSTM1, RAN

## Abstract

To identify metastasis-related proteins in nasopharyngeal carcinoma (NPC), iTRAQ-tagging combined with 2D LC-MS/MS analysis was performed to identify the differentially expressed proteins (DEPs) in high metastatic NPC 5-8F cells and non-metastatic NPC 6-10B cells, and qRT-PCR and Western blotting were used to confirm DEPs. As a result, 101 DEPs were identified by proteomics, and 12 DEPs were selectively validated. We further detected expression of three DEPs (RAN, SQSTM1 and TRIM29) in a cohort of NPC tissue specimens to assess their value as NPC metastatic biomarkers, and found that combination of RAN, SQSTM1 and TRIM29 could discriminate metastatic NPC from non-metastatic NPC with a sensitivity of 88% and a specificity of 91%. TRIM29 and RAN expression level were closely correlated with lymph node and distant metastasis and clinical stage (*P* <0.05) in NPC patients. Finally, a combination of loss-of-function and gain-of-function approaches was performed to determine the effects of TRIM29 on NPC cell proliferation, migration, invasion and metastasis. The results showed that TRIM29 knockdown significantly attenuated while TRIM29 overexpression promoted NPC cell *in vitro* proliferation, migration and invasion and *in vivo* metastasis. The present data first time show that SQSTM1, RAN and TRIM29 are novel potential biomarkers for predicting NPC metastasis, demonstrate that TRIM29 is a metastasis-promoted protein of NPC.

## INTRODUCTION

Nasopharyngeal carcinoma (NPC) is one of the most common malignant tumors in southern China and Southeast Asia. Early metastasis is one of distinctive characteristics of NPC [1]. Although NPC is sensitive to radiotherapy, significant rates of relapse and distant metastasis still occur after therapy, which is a major cause for NPC lethality [2]. The five-year survival rate following combined treatment with radiotherapy and adjuvant cisplatin chemotherapy is 50-60% and the rates of five-year cumulative local relapse and distant metastasis are 20-30 and 20-25%, respectively [3]. However, the molecular mechanism of NPC metastasis is not yet well-defined. To develop better diagnosis and treatment approaches, it is important to identify proteins related to NPC metastasis and understand the molecular basis of NPC metastasis.

Both 5-8F and 6-10B NPC cell lines derived from human NPC low-differentiated squamous cancer cells (SUNE1), which have the same genetic background but not the same metastatic potentials. It has been confirmed that 5-8F possesses high metastatic potential, but 6-10B possesses non- metastatic potential. Therefore, the two cell lines are often used to study the molecular events of NPC metastasis [4, 5].

Proteomics has become a new subject for investigating protein expression and activities. It can comprehensively, dynamically, and quantitatively observe the change of protein expression in the process of tumor development and metastasis [6]. Isobaric tags for relative and absolute quantitation (iTRAQ) in combination with two-dimensional liquid chromatography tandem MS (2D-LC-MS/MS) analysis is emerging as one of the more powerful policy for proteomics methodologies in the search for tumor biomarkers [7–10]. This new approach possesses many advantages compared with the traditional proteomic technologies, such as high throughput, high repeatability, high sensitivity, and high accuracy [7]. Although this approach has been widely applied to screen proteins correlated with NPC [11–15], there are no reports about screening NPC metastasis-associated proteins by using this approach.

The tripartite motif (TRIM) family is composed of multidomain ubiquitin E3 ligases, and characterized by the presence of the N-terminal tripartite motif (RING, B-boxes, and coiled coil). Currently, more than 70 members of TRIM family have been identified in humans. These proteins participate in cell growth and development regulation, and have been implicated in several human diseases such as HIV infection and leukemia [16, 17]. TRIM29 is a member of the tripartite motif (TRIM) family [18]. Several studies have shown that TRIM29 plays an important role in the various types of cancers [19–27]. However, to our best knowledge, the functions of TRIM29 in NPC have never been characterized.

To search biomarkers for early detection of NPC metastasis, in this study iTRAQ tagging followed by 2D LC-MS/MS was performed to identify DEPs between high metastatic 5-8F and non-metastatic 6-10B NPC cells, some DEPs identified by proteomics were selectively validated, the value of three DEPs (RAN, SQSTM and TRIM29) for predicting NPC metastasis was assessed, and the effects of TRIM29 on NPC cell proliferation, migration and invasion were determined.

## RESULTS

### Identification of differentially expressed proteins (DEPs) between 5-8F and 6-10B cell using iTRAQ labeling and 2D-LC-MS/MS

A total of 862 non-redundant proteins were repeatedly identified by iTRAQ labeling and 2D-LC-MS/MS analyses in NPC cells, 79.37% of which were identified with ≥2 peptides matches. The detailed information including information of peptide sequences, protein quantification date, and average iTRAQ ratio for these identified proteins is shown in supplementary Table S-3.

**Figure 1:**
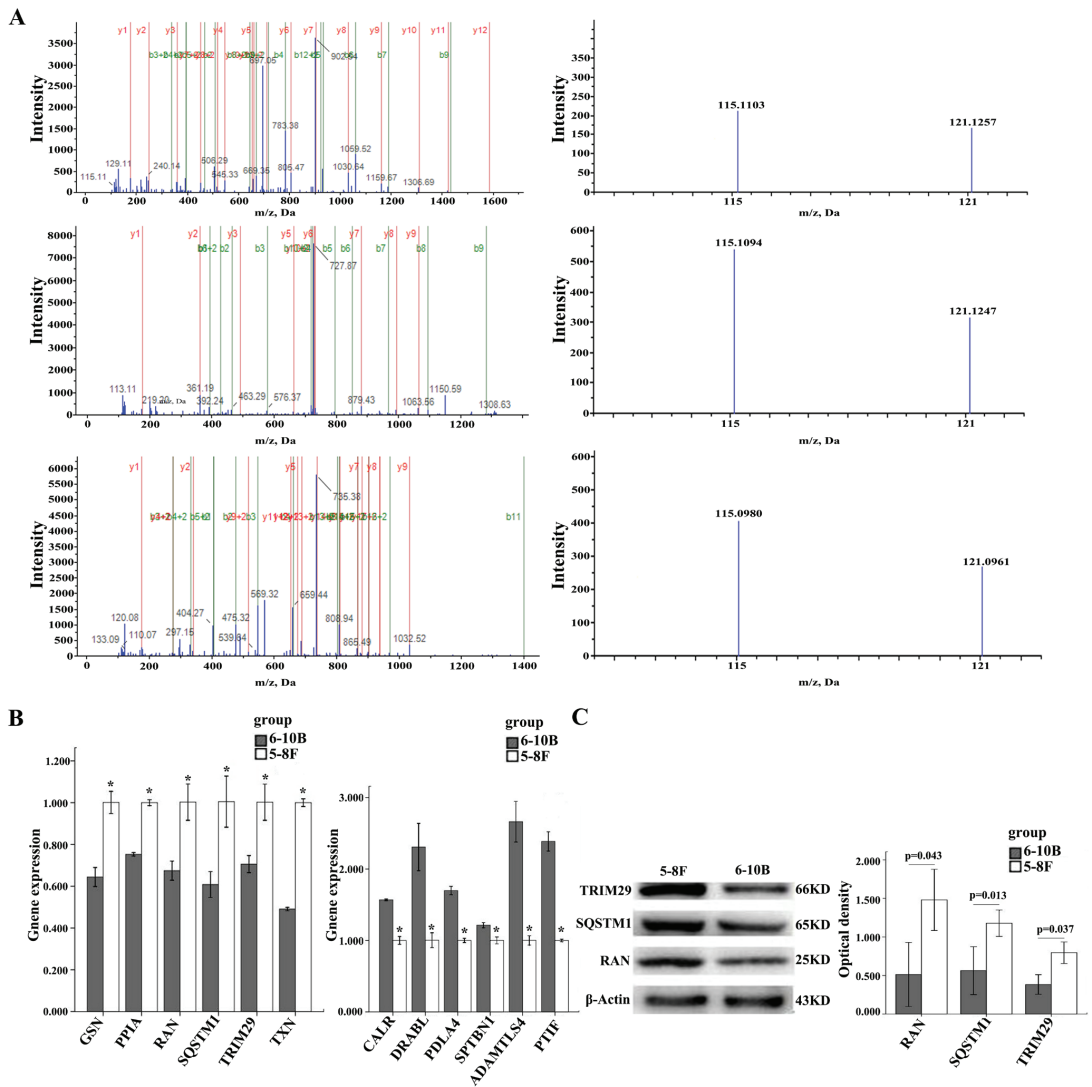
MS/MS spectra of RAN, SQSTM1, and TRIM29 and Expressional changes of TRIM29, RAN, and SQSTM1 in NPC cells **A.** (above) RAN (SNYNFEKPFLWLAR M/Z 697.0439 Z = 3), the sequence SNYNFEKPFLWLAR allows the identification of RAN(right); the released iTRAQ reporter ions provide the relative quantitation of RAN from the two cells evaluated(left). (middle) TRIM29 (SALFAGNEWR M/Z 727.8883 Z = 2), the sequence SALFAGNEWR allows the identification of TRIM29(left); the released iTRAQ reporter ions provide the relative quantitation of TRIM29 from the two cells evaluated(right). (below) SQSTM1 (VAALFPALRPGGFQAHYR M/Z 569.5746 Z = 4), the sequence VAALFPALRPGGFQAHYR allows the identification of SQSTM1(left); the released iTRAQ reporter ions provide the relative quantitation of SQSTM1 from the two cells evaluated(right). 5-8F, labeled with iTRAQ reagent 115; 6-10B, labeled with iTRAQ reagents 121. **B.** The mRNA expressional levels of twelve genes (GPX1, NPM1, CALR, PTGES3, VCP, AIMP1, PPIA, GSN, SQSTM1, TXN, TRIM29, RAN) which were consistent with MS analysis. histogram shows the gene relative expression levels .(left) Expressions of six genes increased in 5-8F compared to 6-10B; ^*^P < 0.05, by Independent sample T test. (right) Expressions of other six genes decreased in 5-8F compared to 6-10B. ^*^P < 0.05, by Independent sample T test; Bars = means ± SD. **C.** A representative result of Western blotting showing the expressions of RAN, SQSTM1, and TRIM29 in the 5-8F and 6-10B (left); histogram shows the expression levels of the three proteins in two kinds of cells as determined by densitometric analysis (right). Actin is used as the internal loading control. Bars = means ± SD; ^*^P < 0.05; by Independent sample T test.

To identify the DEPs related to NPC metastasis, protein expressional profiles between the two cell lines were compared. The proteins which met the following criteria were confidently considered as DEPs: (1) proteins were repeatedly identified by the twice experiments; (2) proteins were identified based on ≥2 peptides; (3) proteins showed an average ratio-fold change ≥1.2 or ≤ 0.8 and the same change trend in the twice experiments between the two cells (*t*-test; *P* < 0.05), as a result, 101 proteins were found to be differentially expressed. The names of the 101 proteins are shown in Supplementary Table S-4. MS/MS spectra used for the identification and quantitation of RAN, SQSTM1, and TRIM29 are shown in Figure 1A.

### Validation of DEPs indentified by quantitative proteomics

Through Uniprot and Pubmed search, 12 different proteins (GSN, PPIA, RAN, SQSTM1, TRIM29, TXN, CALR, DIABLO, PDIA4, SPTBN1, ADAMTSL4, PTRF) identified by MS analysis were chosen in 101 DEPs for verification. QRT-PCR was performed to detect their expression in 5-8F and 6-10B cells. As shown in Figure 1B, the expression changes of their mRNAs are consistent with the findings in MS analysis. In addition, we chose three proteins (RAN, SQSTM1, TRIM29) by Uniprot and Pubmed search for further validation using Western blot. The result showed that the expression of these three proteins was significantly higher in 5-8F cells than that in 6-10B cells (Figure 1C), which are also consistent with the results of MS analysis.

### Value of RAN, SQSTM1 and TRIM29 as biomarkers for predicting NPC metastasis

**Figure 2:**
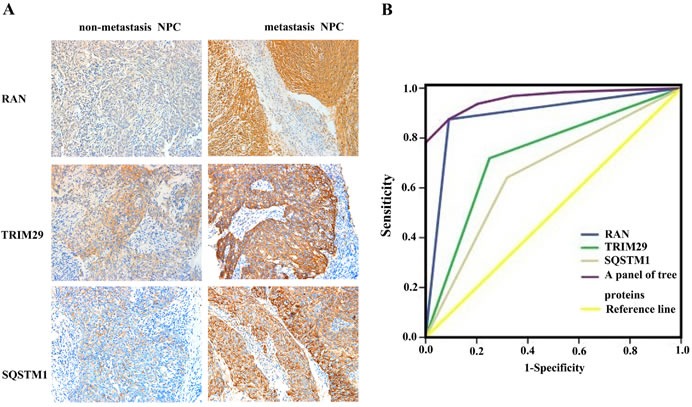
Expressional changes of TRIM29, RAN, and SQSTM1 in NPC tissues and their efficacy in discriminating metastatic NPC from non-metastatic NPC **A.** A representative result of immunohistochemistry shows the expression of RAN, SQSTM1, and TRIM29 in the metastatic NPC and non-metastatic NPC. Original magnification: ×200. **B.** Receiver operating characteristic (ROC) curves of RAN, SQSTM1, TRIM29 in discriminating metastatic NPC from non-metastatic NPC.

To investigate whether the three proteins (RAN, SQSTM1, TRIM29) serve as biomarkers for predicting NPC metastasis, immunohistochemistry was performed to detect the expressional levels of the three proteins in an cohort of formalin-fixed and paraffin-embedded archival tissue specimens including metastatic and non-metastatic NPCs. As shown in Figure 2A and Table 1, the expression levels of all the three proteins were increased in the metastatic NPCs compared with non-metastatic NPCs, which also supports our findings in the NPC cells.

**Table 1:** Expression of RAN, SQSTM1, and TRIM29 expression in 108 nasopharygeal carcinomas

	Non-metastatic NPC	Metastatic NPC
**SQSTM1** High(5-8)	14	41
Low(0-4)	30	23
**RAN** High(5-8)	22	56
Low(0-4)	22	8
**TRIM29** High(5-8)	11	44
Low(0-4)	33	20

*P*<0.01, by Mann-Whitney U test, Non-metastatic NPC *vs.* Metastatic NPC.

The ability of these three proteins in distinguishing metastatic NPC from non-metastatic NPC was analyzed by determining the ROC curves individually and as a panel. The area under the curve (AUC) of these proteins is listed in the Table 2 together with their individual and collective values of merit. When individual protein serves as a biomarker, their sensitivity and specificity are 64-88% and 68-91% in discriminating metastatic NPC from non-metastatic NPC, respectively. As a panel, these proteins achieved a sensitivity of 88% and a specificity of 91% in discriminating metastatic NPC from non-metastatic NPC (Figure 2B; Table 2).

**Table 2:** Receiver operating characteristics of the three proteins from IHC scores, for distinguishing metastatic NPC from non-metastatic NPC (individually and as a panel)

Proteins	Sensitivity	Specificity	PPV	NPV	AUC
RAN	0.88	0.91	0.93	0.83	0.89
TRIM29	0.72	0.75	0.81	0.65	0.73
SQSTM1	0.64	0.68	0.75	0.57	0.66
A panel of three proteins	0.88	0.91	0.93	0.83	0.96

AUC: Area under the curve; NPV: Negative predict value; PPV: Positive predict value.

### The relationship between the expression levels of RAN, SQSTM1 and TRIM29 and clinicopathological characteristics of NPC patients

The relationship between the expression levels of RAN, SQSTM1 and TRIM29 and clinicopathological characteristics in patients with NPC are summarized in Tables 3∼5. We observed that RAN and TRIM29 expression level were closely correlated with lymph node (*P* = 0.000; *P* = 0.000) and distant metastasis (*P* = 0.002; *P* = 0.000), and clinical stage (*P* = 0.036; *P* = 0.041); SQSTM1 expression level was closely correlated with lymph node metastasis (*P* = 0.002), but not correlated with distant metastasis (*P* = 0.073) and clinical stage (*P* = 0.733) in NPC patients. We did not find any significant association of RAN, SQSTM1 and TRIM29 expression with age, sex, and primary lesion size in patients with NPC. The results indicated that high expression of the three proteins enhanced NPC metastasis.

**Table 3:** Association between RAN expression and clinicopathological characteristics in 108 nasopharyngeal carcinomas

Variable		Expression level		
*****n*****	**Low(0-4)**	**High(5-8)**	*****P*****
**Gender**				
Male	72	20	52	1.000
Female	36	10	26	
**Age(y)**				
<50	66	18	48	0.883
≥50	42	12	30	
**Primary tumor(T) stage**				
T1-2	30	11	19	0.201
T3-4	78	19	59	
**lymph node(N) metastasis**				
N0	45	22	23	0.000#
N1-3	63	8	55	
**Distant metastasis(M)**				
M0	77	28	49	0.002#
M1	31	2	29	
**Clinical TNM stage**				
I-II	19	9	10	0.036*
III-IV	89	21	68	

# *P*<0.01 by χ^2^ test, N0 *vs*.N1-3; M0 *vs*.M1.

* *P*<0.05 by χ^2^ test, TNM stage I-II *vs*. III-IV.

**Table 4:** Association between SQSTM1 expression and clinicopathological characteristics in 108 nasopharyngeal carcinomas

Variable		Expression level		
**n**	**Low(0-4)**	**High(5-8)**	*****P*****
**Gender**				
Male	72	35	37	0.892
Female	36	18	18	
**Age(y)**				
<50	66	33	33	0.809
≥50	42	20	22	
**Primary tumor(T) stage**				
T1-2	30	14	16	0.848
T3-4	78	38	40	
**lymph node(N) metastasis**				
N0	45	30	15	0.002#
N1-3	63	23	40	
**Distant metastasis(M)**				
M0	77	42	35	0.073
M1	31	11	20	
**Clinical TNM stage**				
I-II	19	10	9	0.733
III-IV	89	43	46	

# *P*<0.01 by χ^2^ test, N0 *vs*.N1-3.

**Table 5:** Association between TRIM29 expression and clinicopathological characteristics in 108 nasopharyngeal carcinomas

Variable		Expression level		
***n***	**Low(0-4)**	**High(5-8)**	***P***
**Gender**				
Male	72	33	39	0.683
Female	36	18	18	
**Age(y)**				
<50	66	33	33	0.469
≥50	42	18	24	
**Primary tumor(T) stage**				
T1-2	30	15	15	0.720
T3-4	78	36	42	
**lymph node(N) metastasis**				
N0	45	34	11	0.000#
N1-3	63	17	46	
**Distant metastasis(M)**				
M0	77	45	32	0.000#
M1	31	6	25	
**Clinical TNM stage**				
I-II	19	13	6	0.041*
III-IV	89	38	51	

# *P*<0.01 by χ^2^ test, N0 *vs*.N1-3; M0 *vs*.M1.

* *P*<0.05 by χ^2^ test, TNM stage I-II *vs*. III-IV.

### The effects of TRIM29 on NPC cell proliferation, and *in vitro* migration and invasion

**Figure 3:**
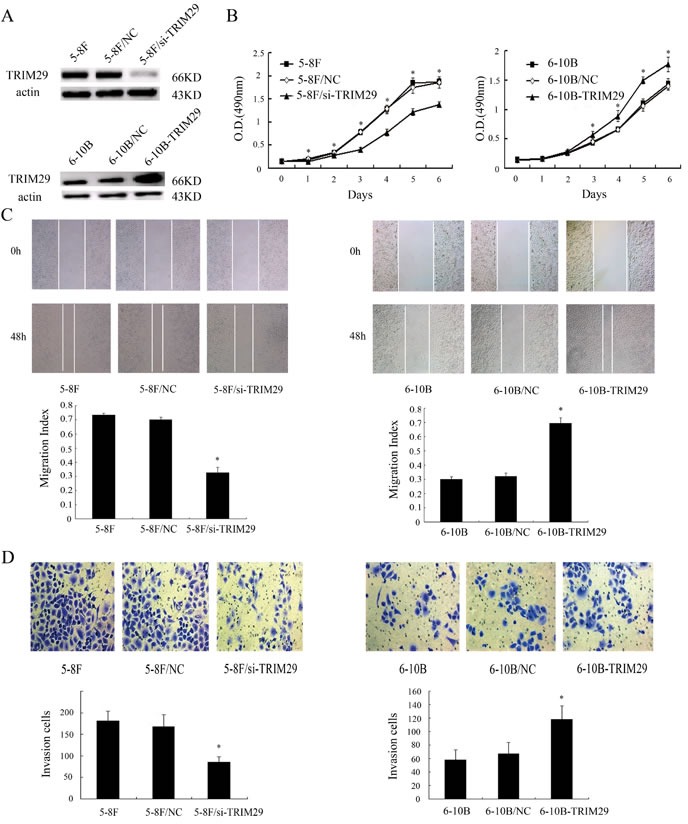
The effects of TRIM29 on NPC cell proliferation, and *in vitro* migration and invasion **A.** (above) A representative result of Western blotting shows the levels of TRIM29 expression in the untransfected (5-8F), empty vector (5-8F/NC) and TRIM29 siRNA plasmid transfected 5-8F cells (5-8F/si-TRIM29). (below) A representative result of Western blotting shows the levels of TRIM29 expression in the untransfected(6-10B), empty vector (6-10B/NC) and TRIM29 expressing plasmid transfected 6-10B cells (6-10B-TRIM29). Actin is used as the internal loading control. **B.** (left) MTT shows knockdown of TRIM29 in 5-8F cells inhabits cell proliferation. (right) MTT shows overexpression of TRIM29 in 6-10B cells promotes cell proliferation. Data were presented as mean±SD(*P < 0.05; by one-way ANOVA). **C.** (left) A representative result of scratch wound-healing assay shows knockdown of TRIM29 in 5-8F cells inhibits cell migration *in vitro*. (right) A representative result of scratch wound-healing assay shows overexpression of TRIM29 in 6-10B cells promotes cell migration *in vitro*. **D.** (left) A representative result of transwell assay shows knockdown of TRIM29 in 5-8F cells inhibits cell invasion *in vitro*. (right) A representative result of transwell assay shows overexpression of TRIM29 in 6-10B cells promotes cell invasion *in vitro*.

The significant increase of TRIM29 expression in the NPC cell and tissues with high metastatic potentials hinted us to investigate its role in NPC cell metastasis both *in vitro* and *in vivo*. We generated 5-8F NPC cell lines with knockdown of TRIM29 and 6-10B NPC cell lines with overexpression of TRIM29 (Figure 3A). MTT assay showed that proliferation of TRIM29 knockdown 5-8F cells significantly decreased compared with control cells (Figure 3B). Conversely, proliferation of TRIM29 overexpression 6-10B cells significantly decreased compared with control cells (Figure 3B). Wound healing and transwell invasion assay showed that TRIM29 knockdown inhibited migration and invasion of 5-8F cells (Figure 3C and 3D), whereas TRIM29 overexpression promoted migration and invasion of 6-10B cells(Figure 3C and 3D). The results suggested that TRIM29 plays a key role in NPC cell migration and invasion *in vitro*.

### The effect of TRIM29 on NPC cell metastasis *in vivo*

**Figure 4:**
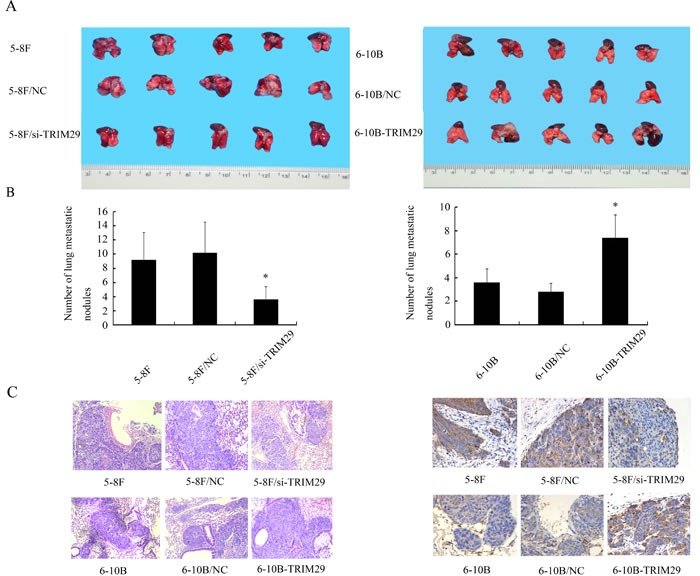
The effect of TRIM29 on NPC cell metastasis *In Vivo* **A.** *In vivo* metastasis assays of NPC cells with TRIM29 expression changes. TRIM29 knockdown 5-8F cells, TRIM29 overexpression 6-10B cells, and their corresponding empty vector-transfected cells,and5-8F cells, 6-10B cells were injected into the tail vein of nude mice, and after 6 weeks the reprentative photography of lung was shown.(left) Knockdown of TRIM29 inhibits metastasis of NPC cells *in vivo*. (right) Overexpression of TRIM29 promotes metastasis of NPC cells *in vivo*. **B.** Histogram of average numbers of lung metastases per mouse. (left) Numbers of lung metastatic nodules in nude mice injected with 5-8F/si-TRIM29 cells is less than 5-8F and 5-8F/NC. (right) Numbers of lung metastatic nodules in nude mice injected with 6-10B-TRIM29 cells is less than injected with 6-10B cells and 6-10B/NC cells. **C.** (left) A representative result of HE shows tumor metastatic condition in 5-8F, 5-8F/NC and 5-8F/si-TRIM29. Original magnification, ×100. (right) A representative result of HE shows tumor metastatic condition in 6-10B, 6-10B/NC and 6-10B-TRIM29. Original magnification, ×100. **D.** (left) A representative result of immunohistochemistry shows the expression of TRIM29 in 5-8F, 5-8F/NC and 5-8F/si-TRIM29. Original magnification, ×400. (right) A representative result of immunohistochemistry shows the expression of TRIM29 in 6-10B, 6-10B/NC and 6-10B-TRIM29. Original magnification, ×400.

To explore the role of TRIM29 in NPC metastasis *in vivo*, we tested the effect of TRIM29 in a xenograft metastasis model in which TRIM29 knockdown 5-8F cells, TRIM29 overexpression 6-10B cells, and their corresponding control cells were used to generate pulmonary metastases in nude mice. As shown in Figure 4, pulmonary metastases generated by TRIM29 knockdown 5-8F cells were significantly less than those generated by control 5-8F cells, whereas pulmonary metastases generated by TRIM29 overexpression 6-10B cells were significantly more than those generated by control 6-10B cells. The results suggested that TRIM29 plays a key role in NPC cell metastasis *in vivo*.

### Gene-ontology, KEGG pathways analysis and PPI of the differential proteins

For an overview of the differential proteins in NPC metastasis, functional analysis including cellular component, molecular functions, biological process and pathways were performed using MAS 3.0 tools. The cellular component category showed that differential proteins are mainly involved in cytoplasm, nucleus, cytosol and mitochondrion. In the molecular function category, DEPs are mainly associated with binding, catalysis, molecular transduction, transport, and molecular structure. The biological process category indicated that the differential proteins are mainly related to signal transduction, transport, cell redox homeostasis, oxidation reduction, RNA splicing and regulation of transcription(Supplementary table 5). An pathway enrichment analysis was conducted by KEGG for 101 differential proteins. By this means, we identified 11 pathways significantly enriched with association signals at the *p* < 0.01 level. These pathways encompassed metabolism, infection, human diseases, ribosome and regulation of actin cytoskeleton. They may play a key role in the process of nasopharyngeal carcinoma metastasis(Supplementary Figure 1). Supplementary Figure 2 shows the PPI generated by STRING 9.5. Differential proteins tightly linked to each other, they interacted and jointly promote the process of nasopharyngeal carcinoma.

## DISCUSSION

Identification of proteins related to NPC metastasis is important in discovery of biomarkers for early warning NPC metastasis. In this study, iTRAQ labeling combined with 2D-LC-MS/MS was used to identify DEPs in the NPC cells with different metastatic potentials. As a result, 101 DEPs were identified, and 12 DEPs were selectively validated. Next, we evaluated the ability of three DEPs (RAN, SQSTM1, TRIM29) for predicting NPC metastasis, and found that panel of these three proteins could greatly distinguish metastatic NPC from non-metastatic NPC with high sensitivity and specificity, suggesting that the three proteins are potential biomarkers for early warning NPC metastasis.

RAN, a GTP-binding nuclear protein, member of small GTPase superfamily, is involved in several important cellular processes, the best known being nucleocytoplasmic transportation [30], Which regulates many cancer-related signaling pathways such as PI3K/Akt/mTORC1 and MEK/ERK, and plays an important role in cancer cell survival and progression [31, 32]. It is overexpressed in various cancers with prognostic significance [33, 34] and its overexpression is correlated with increased aggressiveness of the cancer cells *in vitro* and *in vivo* [35, 36]. Fan H et al. also found that RAN expression increases in colorectal cancer tissues compared with normal colorectal mucosa, and was positively associated with invasion depth, lymph node and distant metastasis, and differentiated degree of the tumors [37]. Our iTRAQ results showed that RAN expression levels increased in NPC tissues with metastasis compared with ones without metastasis, and were positively associated with lymph node and distant metastasis, and clinical stage in NPC patients, supporting that RAN is a metastasis-promoted protein.

SQSTM1, also known as EBI3-associated protein of 60 kDa (EBIAP) or ubiquitin-binding protein p62, regulates a lot of biological processes such as apoptosis, cell differentiation, autophagy, protein localization, and protein ubiquitination [38–42]. It can activate many cancer-related signaling pathways such as NF-kB pathway and MAPK pathway, mTOR pathway, and promote the proliferation and spread of cancer cells [43–49]. SQSTM1 has been identified as a component of inclusion bodies found in various human diseases including HCC and malignant glioma [50, 51]. Furthermore, the loss of SQSTM1 suppressed Ras-induced lung adenocarcinoma [45]. Moreover, dysregulation of NF-kB signal in autophagy- incompetent cells was due, at least in part, to accumulation of SQSTM1, which subsequently enhanced tumorigenesis [52]. Those lines of evidence suggest the involvement of aberrant signals related to p62 accumulation in tumorigenesis. Our results also showed that high SQSTM1 expression positively correlated with NPC metastasis. To our best knowledge, it is first report on SQSTM1′s role in NPC.

**Table 6:** The absorbance values of 5-8F cells after transfecion (490 nm)

cells	Day						
**0**	**1**	**2**	**3**	**4**	**5**	**6**
5-8F	0.145±0.011	0.190±0.028	0.337±0.014	0.772±0.049	1.277±0.093	1.842±0.119	1.876±0.065
5-8F/NC	0.150±0.009	0.206±0.028	0.340±0.035	0.790±0.054	1.287±0.080	1.749±0.129	1.863±0.121
5-8F/si-TRIM29	0.155±0.014	0.148±0.004*#	0.287±0.037*#	0.407±0.046*#	0.777±0.076*#	1.220±0.074*#	1.380±0.062*#
F	1.473	6.765	4.550	66.184	73.833	29.315	42.846
P	0.263	0.011	0.034	0.000	0.000	0.000	0.000

* 5-8F/si-TRIM29 *vs* 5-8F, *P* < 0.05;

# 5-8F/si-TRIM29 *vs* 5-8F/NC, *P* < 0.05.

**Table 7:** The absorbance values of 6-10B cells after transfecion (490 nm)

cells	Day						
**0**	**1**	**2**	**3**	**4**	**5**	**6**
6-10B	0.144±0.009	0.155±0.041	0.252±0.021	0.432±0.027	0.660±0.034	1.099±0.085	1.437±0.092
6-10B/NC	0.142±0.019	0.156±0.034	0.262±0.036	0.456±0.028	0.657±0.037	1.063±0.087	1.395±0.069
6-10B- TRIM29	0.148±0.017	0.170±0.022	0.285±0.038	0.570±0.064*#	0.891±0.096*#	1.497±0.062*#	1.771±0.125*#
F	0.211	0.342	1.548	17.266	19.051	50.934	17.903
P	0.812	0.716	0.247	0.000	0.000	0.000	0.000

* 6-10B-TRIM29 *vs* 6-10B, *P* < 0.05;

# 6-10B-TRIM29 *vs* 6-10B/NC, *P* < 0.05.

TRIM29, also known as Ataxia-telangiectasia group D complementing gene (ATDC), is involved in many human diseases, especially cancer [53]. TRIM29 expression in various tumor types has found that increased expression of TRIM29 is associated with more aggressive forms of disease including bladder [54], colorectal [55], gastric [56], lung [57], and pancreatic cancer [58]. TRIM29 expression in pancreatic cancer cells is 20 times higher than that in normal pancreas epithelial cells, overexpression of TRIM29 in pancreatic cancer lines promoted cell growth *in vitro* and metastatic activity *in vivo* stemming from stimulation of Wnt/β- Catenin/TCF signaling through TRIM29 binding to Dvl-2, a Wnt pathway activator downstream of the Frizzled receptor [19]. Another study found that TRIM29 functions as an oncogene in gastric cancer, the miR-185-TRIM29 axis regulates gastric cancer cell growth and apoptosis through Wnt/β-catenin pathway inactivation *in vitro* [59]. TRIM29 interacts with P53 and antagonizes p53-mediated functions in cancer cells, which increases cell proliferation *via* inhibition of p53 nuclear activities. TRIM29 represses the expression of p53-regulated genes, including p21 and NOXA. Mechanistically, TRIM29 binds p53, and this interaction is potentially fine-tuned by posttranslational acetylation of lysine 116 on TRIM29 [60]. TRIM29 can promote lung cancer proliferation through NF-kB induced up-regulation of cyclin D1 and c-Myc [61]. TRIM29 upregulates MMP-9 to promote lung cancer cell invasion by activating ERK and JNK pathways [62]. However, there is not report on the function of this protein in NPC. We found and confirmed that TRIM29 expression increased in NPC cells and tissues with high metastatic potentials, and high TRIM29 expression were correlated with lymph node and distant metastasis of PCR. To further investigate the effects of TRIM29 on NPC cell invasion and metastasis, we established NPC cell lines with stable TRIM29 knockdown or overexpression. We found that TRIM29 knockdown significantly attenuated while TRIM29 overexpression promoted NPC cell *in vitro* proliferation, migration and invasion and *in vivo* metastasis. Taken together, our results demonstrated that TRIM29 is a metastasis promoter protein of NPC. Furthermore, we will do further study of molecular mechanisms of NPC metastasis.

To get more insight on the biological significance of the differentially expressed proteins in NPC metastasis process, gene-ontology, KEGG pathways analysis and PPI were performed on 101 differential proteins. Cellular component, molecular functions, biological process which these proteins were enriched were performed by GO analysis. KEGG pathway analysis revealed that the differentially expressed proteins are involved in cancer-associated signaling pathways.

In summary, we identified a total of 101 DEPs in NPC cells with different metastatic potentials by iTRAQ-labeling combined with 2D-LC-MS/MS and found that the panel of the three proteins (RAN, SQSTM1 and TRIM29) could serve as novel potential biomarkers for predicting NPC metastasis, and high TRIM29 expression promoted NPC cell *in vitro* proliferation, migration and invasion and *in vivo* metastasis. These findings reported here could have potential clinical value in early warning NPC metastasis and also provide valuable information for further study of molecular mechanisms that govern NPC metastasis.

## MATERIALS AND METHODS

### Cell lines and tissue samples

High metastatic NPC 5-8F and non-metastatic NPC 6-10B cell lines were maintained in RPMI-1640 medium supplemented with 10% FBS in a humidified chamber with 5% CO_2_ at 37°C. The one hundred and eight formalin-fixed and paraffin-embedded archival NPC tissue specimens (64 NPCs with metastasis and 44 NPCs without metastasis) between Jan 2007 and Dec 2009 were obtained from the First Xiangya Hospital of Central South University and the First Hospital of Chenzhou City (Chenzhou, China) at the time of diagnosis before any therapy. On the basis of the 1978 WHO classification, all tumors were histopathologically diagnosed as poorly differentiated squamous cell carcinomas (WHO type III) [28]. The clinical stage of all the patients was classified according to the 1992 NPC staging system of China [29]. The clinicopathologic features of the patients used in the present study are shown in supplementary Table S-1.

### Protein extraction, digestion and labeling with iTRAQ reagents

Cells were dissolved in lysis buffer (7 M urea, 2M thiourea, 65 mM dithiothreitol, 0.1 mM phenylmethylsulfonyl fluoride) at 4°C for 1 h, and then centrifuged at 12,000 rpm for 30 min at 4°C. The supernatant was collected, and the protein concentration was determined by 2D Quantification kit (Applied Biosystems). Trypsin digestion and iTRAQ labeling were performed according to the manufacturer's protocol (Applied Biosystems, Foster City, CA). Briefly, 100μg protein of each sample was reduced and alkylated, then digested overnight at 37°C with trypsin (mass spectrometry grade; Promega, Madison, WI) and labeled with iTRAQ™ Reagents(Applied Biosystems) as follows: 6-10B, iTRAQ Reagent 121; 5-8F, iTRAQ Reagents 115 (the other channels for other samples). Two labeled digests were mixed and dried.

### Off-line 2D-LC-MS/MS

The mixed peptides were fractionated by strong cation exchange chromatography on a 20AD HPLC system (Shimadzu) using a polySULFOETHYL column (2.1 100 mm, 5 m, 300Å; The Nest Group Inc.). Briefly, the mixed peptides were desalted with Sep-Pak Cartridge (Waters, Milford, MA), diluted with the loading buffer (10 mM KH2PO4 in 25% acetonitrile, pH 2.6), and loaded onto the column. Buffer A was identical in composition to the loading buffer, and buffer B was same as buffer A except that it contained 350 mM KCl. Separation was performed using a linear binary gradient of 0-88% buffer B in buffer A at a flow rate of 200μl/min for 60 min. The absorbance at 214 nm and 280 nm was monitored, and a total of 26 strong cation exchange fractions were collected along the gradient.

Each strong cation exchange fraction was dried down, dissolved in buffer C (5% acetonitrile, 0.1% formic acid), and analyzed on Qstar XL (Applied Biosystems). Briefly, peptides were separated on a reverse-phase (RB) column (ZORBAX 300SB-C18 column, 5 μm, 300Å, 0.1×15 mm; MicroMass) using a 20AD HPLC system (Shimadzu). The HPLC gradient was 5-35% buffer D (95% acetonitrile, 0.1% formic acid) in buffer C at a flow rate of 300 nL/min for 100 min. Survey scans were acquired from 400-1800 with up to four precursors selected for MS/MS from m/z 100-2000 using a dynamic exclusion of 30S. The iTRAQ labeled peptides fragmented under collision-induced dissociation conditions to give reporter ions at 115.1, 121.1 Th. The ratios of peak areas of the iTRAQ reporter ions reflect the relative abundances of the peptides and consequently, the proteins in the samples. Larger sequence-information-rich fragment ions were also produced under these MS/MS conditions and gave the identity of the protein from which the peptide originated. iTRAQ labeling followed by 2D-LC-MS/MS analysis was repeated twice(different protein lysates) to diminish the effect of experimental variation in the results of a proteomics analysis.

### Data analysis

The software used for data acquisition was Analyst QS 1.1 (Applied Biosystems). The software used for protein identification and quantitation was ProteinPilot™ 4.2 (Applied Biosystems) with the integrated Paragon™ search algorithm and Pro Group™ algorithm (Revision Number: 3.0.0.0, 113442; Applied Biosystems). Identified proteins were grouped by the software to minimize redundancy. All the peptides used for the calculation of protein ratios were unique to the given protein or proteins within the group, and peptides that were common to other isoforms or proteins of the same family were ignored. The protein confidence threshold cutoff is 1.3 (unused ProtScore) with at least one peptide with 95% confidence. The average iTRAQ ratios from the twice experiments were calculated for each protein.

### QRT-PCR

Total RNA were extracted from cells using Trizol reagent (Invitrogen) according to the manufacturer's instructions. 2 μg of total RNA was reversely transcribed for cDNA using the reverse transcription (RT) kit (Promega) and Oligo dT primer according to the manufacturer's instruction. The RT products were amplified by real-time PCR using QuantiFast SYBR green PCR kit (Qiagen) according to the manufacturer's instruction, and GAPDH was used as the internal control to normalize the expression levels of different genes. QRT-PCR was performed on the ABI Gene Amp PCR System 9700 (ABI). The primers used for the amplification of the indicated genes are listed in Supplementary Table S-2.

### Western blotting

Cells were lysed in RIPA buffer. An equal amount of protein in each sample was mixed with Laemmli buffer and subjected to sodium dodecyl sulfate-polyacrylamide gel electrophoresis (SDS-PAGE) separation, followed by blotting onto a polyvinylidene difluoride (PVDF) membrane (Millipore). Blots were incubated with primary anti-RAN antibody (1:1000; Proteintech), anti-TRIM29 antibody (1:200; Santa Cruz), anti-SQSTM1 antibody (1:100;Santa Cruz), or anti-actin antibody (1:2000; AURAGENE, China) overnight at 4°C, followed by incubation with goat anti-rabbit antibody (1:6000; Jackson Immuno Research) or goat anti-mouse antibody (1:3000; Jackson Immuno Research) for 1 h at room temperature. The signal was visualized with Luminata Crescendo Western HRP Substrate (Millipore, USA) and quantitated by densitometry using ImageQuant image analysis system (Storm Optical Scanner, Molecular Dynamics, Sunnyvale, CA).

### Immunohistochemistry and evaluation of staining

Immunohistochemistry was performed on formalin-fixed and paraffin-embedded tissue sections using a standard. Briefly, 4 μm of tissue sections were deparaffinized, rehydrated, and treated with an antigen retrieval solution (10 mmol/L sodium citrate buffer, pH 6.0). The sections were incubated with anti-RAN (1:100), anti-TRIM29 (1:50), or anti-SQSTM1 (1:50) antibody overnight at 4°C and then were incubated with 1:1000 dilution of biotinylated secondary antibody followed by avidin-biotin peroxidase complex (DAKO) according to the manufacturer's instructions. Finally, tissue sections were incubated with 3, 3′-Diaminobenzidine (Sigma) until a brown color was developed and counterstained with Harris modified hematoxylin. In negative controls, primary antibodies were omitted.

Immunostaining was blindly evaluated by two pathologists in an effort to provide a consensus on staining patterns by light microscopy. A quantitative score was performed by adding the score of staining extent and the score of staining intensity for each case to assess the expression levels of the proteins. Staining intensity was categorized: negative as 0, bordering as 1, weak as 2, moderate as 3, and strong as 4. The percentage of stained cells was categorized as 0–25% of stained cells = 1, 26–50% = 2, 51–75% = 3, and 76–100% =4. The staining score (ranging from 0-8) for each tissue was calculated by adding the area score and the intensity score. A combined staining score of ≤4 was considered to be low expression; and a score of >4 was considered to be high expression.

### Establishment of NPC Cell Lines with overexpression and knockdown of TRIM29

To generate NPC cell lines with TRIM29 knockdown, GV102-TRIM29-shRNA vector and empty vector GV102 (Genechem, shanghai, China) were transfected into 5-8F cells using Lipofectamin 2000 (Invitrogen, USA) according to the manufacturer's instructions, respectively. To generate NPC cell lines with TRIM29 overexpression, GV143-TRIM29 expression vector and empty vector GV143 (Genechem) were transfected into the 6-10B cells using Lipofectamin 2000, respectively. Cells were selected using neomycin for 2 weeks, and NPC cell lines with stable overexpression and knockdown of TRIM29 and their respective control cell lines were obtained.

### Cell proliferation assay

The cells in RPIM-1640 medium containing 10% fetal calf serum were plated at 2×10^3^ cells per well in 96-well tissue culture plates, and grew for 6 days. Every 24 h, 20 μl of MTT (5 mg/ml; Sigma) was added to wells, and the medium was removed after 4 h of incubation. 250 μl of dimethylsulfoxide(DMSO) was added to each well for 10 min at room temperature. The absorbance of each well was read with a Bio-Tek Instruments EL310 Microplate Autoreader at 490 nm. MTT assay was performed three times in triplicate.

### Wound healing assay

Cell migration was determined by scratch wound-healing assay. Briefly, cells were grown in RPMI 1640 medium containing 10% FBS overnight to confluence in a 6-well plate. Monolayers of cells were wounded by dragging a pipette tip. Cells were washed to remove cellular debris and allowed to migrate for 24-48h. Images were taken at 0, 24 and 48h after wounding under the inverted microscope.

### Cell invasion assay

Invasion assays were performed in 24-well 8-mm pore size transwell chambers precoated with Matrigel (Corning) according to the manufacturer's instruction. The upper chamber was filled with 1×10^5^ cells in RPMI 1640 medium containing 0.5% FBS. The lower chamber was filled with RPMI 1640 medium containing 10% FBS as a chemoattractant. After incubation at 37°C for 48h, cells were fixed with 4% paraformaldehyde and stained with 0.5% crystal violet. Cells migrating through the Matrigel and the pores of the filter were counted from four random microscopic fields.

### Experimental lung metastasis in nude mice

Nude male mice that were 4 weeks old were obtained from the Laboratory Animal Center of Central South University (Changsha, China) and were maintained under specific pathogen-free conditions. For experimental lung metastasis, mice (*n* = 6 each group) were injected intravenously with 1×10^7^ cells/mouse *via* the tail vein. Six weeks after injection, mice were sacrificed by cervical dislocation, and lungs were removed, weighed, and embedded in paraffin for hematoxylin and eosin (H.E.) and immunohistochemical staining. Lung metastases were detected in paraffin-embedded tissue sections stained with H.E. staining.

### Statistical analysis

Statistical analysis was performed using SPSS 17.0 software. Difference of RAN, SQSTM1, and TRIM29 protein expression between the two different kinds of tissues (metastatic NPC *vs*. non-metastatic NPC) was analyzed using Mann-Whitney U test. Moreover, the three proteins were individually, and as a panel, assessed for its ability to discriminate metastatic NPC from non-metastatic NPC lesions by evaluating its ROC curve based on the immunohistochemistry scores. Sensitivity, specificity, positive predictive value, and negative predictive value of the three proteins were calculated individually and as a panel. The relationship between RAN, SQSTM1, and TRIM29 expression and the clinicopathological characteristics in patients with NPC was analyzed using Mann-Whitney U test. Student's *t*-test was used for comparisons between two groups and one-way ANOVA was used for comparisons among more than two groups. A two-sided *P* < 0.05 was considered significant.

### Ethics statement

This study was approved by the Institute Research Ethics Committee of Central South University, China. No informed consent (written or verbal) was obtained for use of retrospective tissue samples from the patients in the study, since most of the patients were deceased and informed consent was not deemed necessary and waived by the Ethics Committee. All animal experiments were undertaken in accordance with the Guide for the Care and Use of Laboratory Animals of Central South University, with the approval of the Scientific Investigation Board of Central South University.

### Bioinformatics analysis

Differential expression proteins were annotated by GO using MAS 3.0 (http://bioinfo.capitalbio.com/mas3/). GO terms with computed p values less than 0.05 were considered as significantly enriched. KEGG pathway analysis was performed with the protein-protein interaction network of the differential proteins using Cytoscape (V2.8.2) with the ClueGO v1.4 plugin. A protein-protein interaction network(PPI) was constructed using Search Tool for the Retrieval of Interacting Genes/Proteins (String 9.05), in which the differential proteins served as a bait, and the proteins have a direct experimental interaction with the bait proteins in the databases. KEGG pathway was considered statistically significant when the corrected *p* value was less than 0.01.

## SUPPLEMENTARY MATERIALS FIGURES AND TABLES






