# Supplementary material for: Identification of nasopharyngeal carcinoma metastasis-related biomarkers by iTRAQ combined with 2D-LC-MS/MS
Source: Oncotarget. 2016 Apr 27;7(23):34022–37. doi: 10.18632/oncotarget.9067 (PMC5085135; doi:10.18632/oncotarget.9067)
Supplement: Supplementary file 1 [file oncotarget-07-34022-s001.pdf]

Identification of nasopharyngeal carcinoma metastasis-related biomarkers by iTRAQ combined with 2D-LC-MS/MS

Supplementary Material

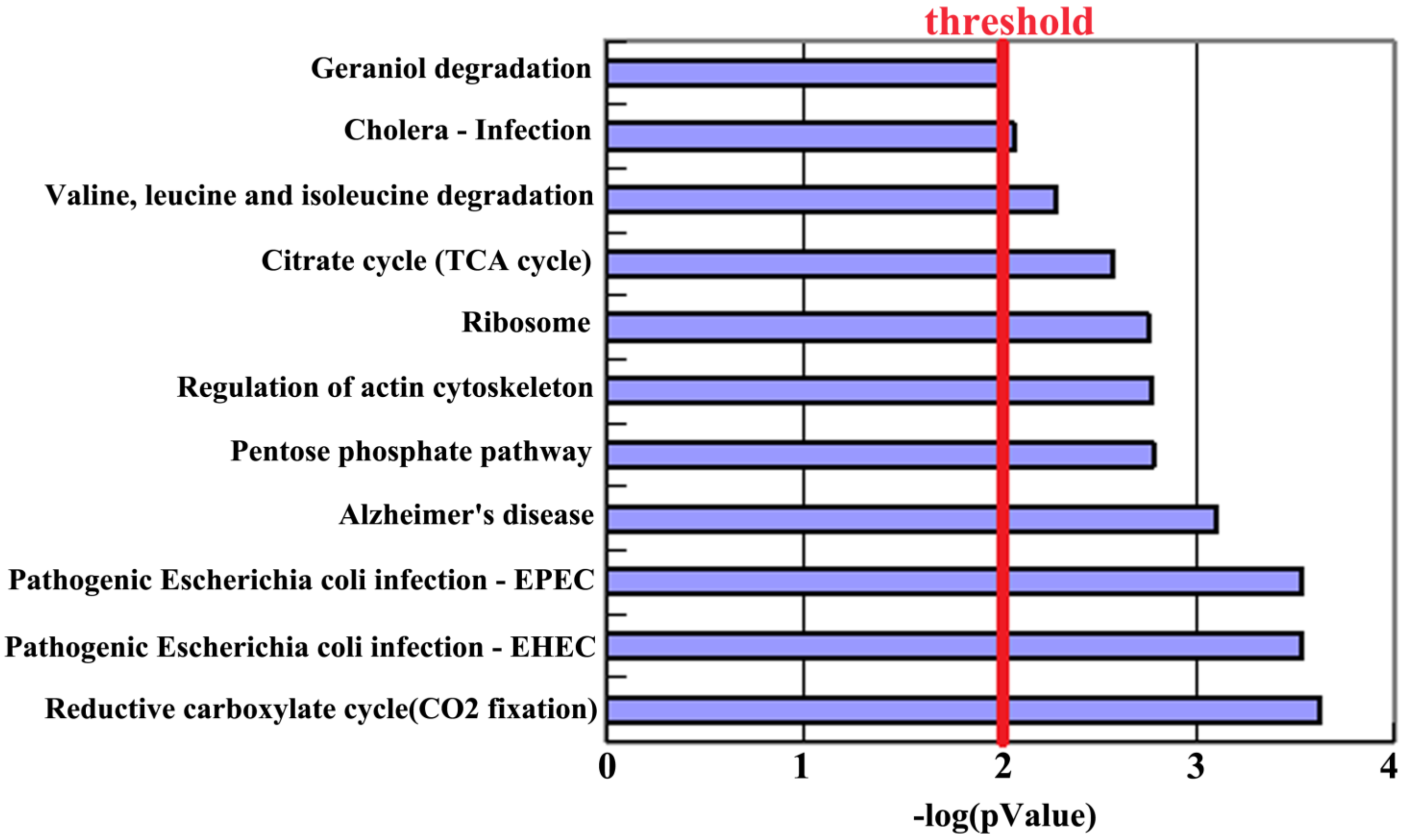

Fig S1 Pathways enrichment analysis of proteins differentially expressed in 5-8F as compared to 6-10B .

each bar represents a significantly enriched pathway as determined using the multiple test corrected Fisher's Exact Test P-value. The P-value is depicted as–log10 (BH P value) on primary X-axis(P<0.01,-log（p）>2）.

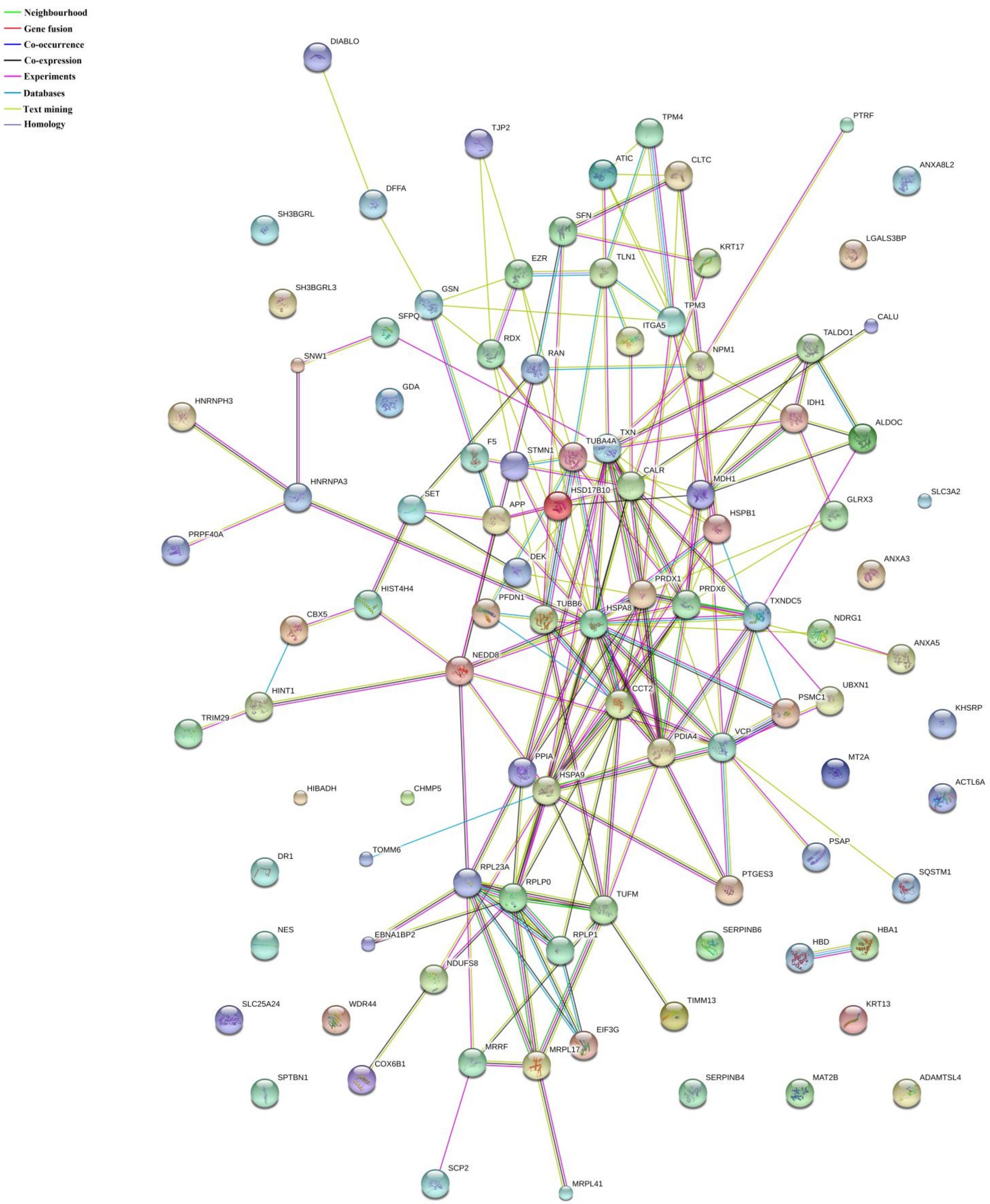

Supplementary Fig S2 Interaction network diagram of DEP in 5-8F compared to 6-10B.

Supplementary Table S1. The clinicopathological parameters of 108 patients with nasopharygeal carcinoma

Variable	No. of patients	%
**Gender**		
Male	72	66.67
Female	36	33.33
**Age**		
≥50	42	38.89
<50	66	61.11
**Primary tumor(T) stage**		
T1-2	30	27.78
T3-4	78	72.22
**Lymph node(N) metastasis**		
N0	45	41.67
N1-3	63	58.33
**Distant metastasis(M)**		
M0	77	71.30
M1	31	28.70
**Clinical stage**		
I-II	19	17.59
III-IV	89	82.41

Supplementary Table S2. The primers used for the amplification of the twelve genes by qRT-PCR

No.	Gene name	GenBank Accession No.	Primer sequence	Product size(bp)
1	GSN	NM_000177	F: GGTGTGGCATCAGGATTCAAG	199
			R: TTTCATACCGATTGCTGTTGGA	
2	PPIA	NM_021130	F: CAGACAAGGTCCCAAAGACAG	298
			R: TTGCCATCCAACCACTCAGTC	
3	RAN	NM_001300	F: TGGTTGGTGATGGTGGTA	137
			R: CTTAATAGGTCCTCTGTTGGT	
4	SQSTM1	NM_003900	F: GACTACGACTTGTGTAGCGTC	139
			R: AGTGTCCGTGTTTCACCTTCC	
5	TRIM29	NM_012101	F: GCATAGCATCAGCGACTC	220
			R: GTTCCTCTCAATGAAGTTACG	
6	TXN	NM_003329	F: AAGAAGGGACAAAAGGTGG	239
			R: GCTATTCAGACATGAGACGGT	
7	CALR	NM_004343	F: CTCTGTCGGCCAGTTTCGAG	159
			R: TGTATTCTGAGTCTCCGTGCAT	
8	DIABLO	NM_138930	F: TCAGAGATGGCAGCAGAAG	234
			R: CCCTCAATCCTCACGCAG	
9	PDIA4	NM_004911	F: CCACCGCAGAAACAGACCT	97
			R: GGGCCGTTGTAGTCATAAGGC	
10	SPTBN1	NM_178313	F: AATTTGCCAATTCACTGGTCGG	167
			R: CCGGGGCATGTAGACCTTC	
11	ADAMTSL4	NM_019032	F: GCTATTCCGTCCCCTACTCCA	130
			R: CAGTTTCAGGGCTTAGAGGTTC	
12	PTRF	NM_012232	F: GGGCCGTAGACCAGATCCA	140
			R: CTTGCTCACCGTATTGCTCGT	

Supplementary Table 4. Differentially expressed proteins (gene) during human nasopharyngeal carcinoma metastasis process summary table

No	Accession #	Name	Ratio(5-8F/6-10B)
1	sp|P11142|HSP7C_HUMAN	Heat shock cognate 71 kDa protein	1.51356101
2	sp|P55072|TERA_HUMAN	Transitional endoplasmic reticulum ATPase	1.737800956
3	sp|P27797|CALR_HUMAN	Calreticulin	0.765596628
4	sp|P06748|NPM_HUMAN	Nucleophosmin	1.406048059
5	sp|P62937|PPIA_HUMAN	Peptidyl-prolyl cis-trans isomerase A	1.803017974
6	sp|P38646|GRP75_HUMAN	Stress-70 protein, mitochondrial	1.706081986
7	sp|P67936|TPM4_HUMAN	Tropomyosin alpha-4 chain	0.704693079
8	sp|P13667|PDIA4_HUMAN	Protein disulfide-isomerase A4	0.220800504
9	sp|P15311|EZRI_HUMAN	Ezrin	1.158776999
10	sp|O43852|CALU_HUMAN	Calumenin	0.602559626
11	sp|P78371|TCPB_HUMAN	T-complex protein 1 subunit beta	1.599557996
12	sp|P08758|ANXA5_HUMAN	Annexin A5	0.642687678
13	sp|P31947|1433S_HUMAN	14-3-3 protein sigma	1.599557996
14	sp|Q01105|SET_HUMAN	Protein SET	0.619441092
15	sp|Q04695|K1C17_HUMAN	Keratin, type I cytoskeletal 17	1.940886021
16	sp|P23246|SFPQ_HUMAN	Splicing factor, proline- and glutamine-rich	0.751622915
17	sp|P04792|HSPB1_HUMAN	Heat shock protein beta-1	0.816582382
18	sp|Q06830|PRDX1_HUMAN	Peroxiredoxin-1	1.180320978
19	sp|Q6NZI2|PTRF_HUMAN	Polymerase I and transcript release factor	0.478630096
20	sp|Q9Y490|TLN1_HUMAN	Talin-1	0.35318321
21	sp|P49773|HINT1_HUMAN	Histidine triad nucleotide-binding protein 1	1.836537957
22	sp|P10599|THIO_HUMAN	Thioredoxin	1.51356101

No	Accession #	Name	Ratio(5-8F/6-10B)
23	sp|Q8NBS9|TXND5_HUMAN	Thioredoxin domain-containing protein 5	0.691830993
24	sp|P06396|GELS_HUMAN	Gelsolin	2.202263951
25	sp|P30041|PRDX6_HUMAN	Peroxiredoxin-6	3.133285999
26	sp|P13646|K1C13_HUMAN	Keratin, type I cytoskeletal 13	0.847227395
27	sp|P62805|H4_HUMAN	Histone H4	0.457088202
28	sp|P35241|RADI_HUMAN	Radixin	1.599557996
29	sp|P31942|HNRH3_HUMAN	Heterogeneous nuclear ribonucleoprotein H3	1.599557996
30	sp|P16949|STMN1_HUMAN	Stathmin	1.923092008
31	sp|Q13501|SQSTM_HUMAN	Sequestosome-1	6.025596142
32	sp|Q15185|TEBP_HUMAN	Prostaglandin E synthase 3	1.30617094
33	sp|P07602|SAP_HUMAN	Proactivator polypeptide	0.801678121
34	sp|Q01082|SPTB2_HUMAN	Spectrin beta chain, brain 1	0.758577585
35	sp|P31939|PUR9_HUMAN	Bifunctional purine biosynthesis protein PURH	1.180320978
36	sp|O00273|DFFA_HUMAN	DNA fragmentation factor subunit alpha	1.270573974
37	sp|P49411|EFTU_HUMAN	Elongation factor Tu, mitochondrial	1.29419601
38	sp|P09972|ALDOC_HUMAN	Fructose-bisphosphate aldolase C	0.591561615
39	sp|Q13069|GAGE5_HUMAN	G antigen 5	0.801678121
40	sp|P05388|RLA0_HUMAN	60S acidic ribosomal protein P0	3.837071896
41	sp|P12429|ANXA3_HUMAN	Annexin A3	0.704693079
42	sp|Q13573|SNW1_HUMAN	SNW domain-containing protein 1	1.51356101
43	sp|Q92597|NDRG1_HUMAN	Protein NDRG1	0.660693526
44	sp|Q9BUF5|TBB6_HUMAN	Tubulin beta-6 chain	0.801678121
45	sp|O75821|EIF3G_HUMAN	Eukaryotic translation initiation factor 3 subunit G	2.355048895
46	sp|Q14134|TRI29_HUMAN	Tripartite motif-containing protein 29	2.466038942

No	Accession #	Name	Ratio(5-8F/6-10B)
47	sp|Q9Y5L4|TIM13_HUMAN	Mitochondrial import inner membrane translocase subunit Tim13	0.717794299
48	sp|Q9NZL9|MAT2B_HUMAN	Methionine adenosyltransferase 2 subunit beta	1.19124198
49	sp|P31937|3HIDH_HUMAN	3-hydroxyisobutyrate dehydrogenase, mitochondrial	0.787045777
50	sp|Q9NZZ3|CHMP5_HUMAN	Charged multivesicular body protein 5	1.995262027
51	sp|Q04323|UBXN1_HUMAN	UBX domain-containing protein 1	0.515228629
52	sp|Q15843|NEDD8_HUMAN	NEDD8	1.258924961
53	sp|Q92945|FUBP2_HUMAN	Far upstream element-binding protein 2	0.698232412
54	sp|P37837|TALDO_HUMAN	Transaldolase	1.202263951
55	sp|P62750|RL23A_HUMAN	60S ribosomal protein L23a	0.608134985
56	sp|Q5VT79|AXA82_HUMAN	Annexin A8-like protein 2	0.564936996
57	sp|P14854|CX6B1_HUMAN	Cytochrome c oxidase subunit 6B1	0.660693526
58	sp|P69905|HBA_HUMAN	Hemoglobin subunit alpha	1.318256974
59	sp|O76003|GLRX3_HUMAN	Glutaredoxin-3	1.485936046
60	sp|P62191|PRS4_HUMAN	26S protease regulatory subunit 4	0.758577585
61	sp|P35659|DEK_HUMAN	Protein DEK	1.30617094
62	sp|P02042|HBD_HUMAN	Hemoglobin subunit delta	0.654636085
63	sp|P06753|TPM3_HUMAN	Tropomyosin alpha-3 chain	0.597035289
64	sp|P05067|A4_HUMAN	Amyloid beta A4 protein	1.213389039
65	sp|Q9NRX2|RM17_HUMAN	39S ribosomal protein L17, mitochondrial	0.824138105
66	sp|O75368|SH3L1_HUMAN	SH3 domain-binding glutamic acid-rich-like protein	0.847227395
67	sp|P40925|MDHC_HUMAN	Malate dehydrogenase, cytoplasmic	2.582259893
68	sp|P05386|RLA1_HUMAN	60S acidic ribosomal protein P1	1.213389039
69	sp|P02795|MT2_HUMAN	Metallothionein-2	1.419057012
70	sp|Q9NR28|DBLOH_HUMAN	Diablo homolog, mitochondrial	0.594328213

No	Accession #	Name	Ratio(5-8F/6-10B)
71	sp|O75400|PR40A_HUMAN	Pre-mRNA-processing factor 40 homolog A	1.28233099
72	sp|Q6NUK1|SCMC1_HUMAN	Calcium-binding mitochondrial carrier protein SCaMC-1	0.816582382
73	sp|Q00610|CLH1_HUMAN	Clathrin heavy chain 1	0.660693526
74	sp|Q9UDY2|ZO2_HUMAN	Tight junction protein ZO-2	1.258924961
75	sp|P62826|RAN_HUMAN	GTP-binding nuclear protein Ran	1.656818008
76	sp|P12259|FA5_HUMAN	Coagulation factor V	0.625172675
77	sp|P08648|ITA5_HUMAN	Integrin alpha-5	0.801678121
78	sp|P08195|4F2_HUMAN	4F2 cell-surface antigen heavy chain	0.474242002
79	sp|Q99714|HCD2_HUMAN	3-hydroxyacyl-CoA dehydrogenase type-2	0.758577585
80	sp|P48594|SPB4_HUMAN	Serpin B4	0.666806817
81	sp|P48681|NEST_HUMAN	Nestin	1.706081986
82	sp|Q99848|EBP2_HUMAN	Probable rRNA-processing protein EBP2	0.672976673
83	sp|O96019|ACL6A_HUMAN	Actin-like protein 6A	1.258924961
84	sp|P35237|SPB6_HUMAN	Serpin B6	0.809095919
85	sp|P45973|CBX5_HUMAN	Chromobox protein homolog 5	1.976969957
86	sp|O00217|NDUS8_HUMAN	NADH dehydrogenase [ubiquinone] iron-sulfur protein 8, mitochondrial	0.597035289
87	sp|P67870|CSK2B_HUMAN	Casein kinase II subunit beta	1.318256974
88	sp|Q8IXM3|RM41_HUMAN	39S ribosomal protein L41, mitochondrial	0.524807513
89	sp|Q96E11|RRFM_HUMAN	Ribosome-recycling factor, mitochondrial	0.48305881
90	sp|O75874|IDHC_HUMAN	Isocitrate dehydrogenase [NADP] cytoplasmic	0.539510608
91	sp|Q96B49|TOM6_HUMAN	Mitochondrial import receptor subunit TOM6 homolog	1.527565956
92	sp|Q6UY14|ATL4_HUMAN	ADAMTS-like protein 4	0.73790431
93	sp|Q9Y2T3|GUAD_HUMAN	Guanine deaminase	0.73790431
94	sp|Q9H299|SH3L3_HUMAN	SH3 domain-binding glutamic acid-rich-like protein 3	0.549540877

No	Accession #	Name	Ratio(5-8F/6-10B)
95	sp|O60925|PFD1_HUMAN	Prefoldin subunit 1	0.855066717
96	sp|Q08380|LG3BP_HUMAN	Galectin-3-binding protein	1.485936046
97	sp|P51991|ROA3_HUMAN	Heterogeneous nuclear ribonucleoprotein A3	0.685488224
98	sp|P68366|TBA4A_HUMAN	Tubulin alpha-4A chain	1.19124198
99	sp|Q01658|NC2B_HUMAN	Protein Dr1	0.492039502
100	sp|Q5JSH3|WDR44_HUMAN	WD repeat-containing protein 44	0.704693079
101	sp|P22307|NLTP_HUMAN	Non-specific lipid-transfer protein	1.786488056
